# Alignment between PIN1 Polarity and Microtubule Orientation in the Shoot Apical Meristem Reveals a Tight Coupling between Morphogenesis and Auxin Transport

**DOI:** 10.1371/journal.pbio.1000516

**Published:** 2010-10-19

**Authors:** Marcus G. Heisler, Olivier Hamant, Pawel Krupinski, Magalie Uyttewaal, Carolyn Ohno, Henrik Jönsson, Jan Traas, Elliot M. Meyerowitz

**Affiliations:** 1 Division of Biology, California Institute of Technology, Pasadena, California, United States of America; 2 INRA, CNRS, ENS, Université de Lyon, Lyon Cedex, France; 3 Computational Biology and Biological Physics Group, Department of Theoretical Physics, Lund University, Lund, Sweden; University of York, United Kingdom

## Abstract

Morphogenesis during multicellular development is regulated by intercellular signaling molecules as well as by the mechanical properties of individual cells. In particular, normal patterns of organogenesis in plants require coordination between growth direction and growth magnitude. How this is achieved remains unclear. Here we show that in *Arabidopsis thaliana*, auxin patterning and cellular growth are linked through a correlated pattern of auxin efflux carrier localization and cortical microtubule orientation. Our experiments reveal that both PIN1 localization and microtubule array orientation are likely to respond to a shared upstream regulator that appears to be biomechanical in nature. Lastly, through mathematical modeling we show that such a biophysical coupling could mediate the feedback loop between auxin and its transport that underlies plant phyllotaxis.

## Abstract 2

Imaging and computational modeling of the *Arabidopsis* shoot meristem epidermis suggests that biomechanical signals coordinately regulate auxin efflux carrier distribution and microtubule patterning to orchestrate the extent and directionality of growth.

## Author Summary

The proper development of plant organs such as leaves or flowers depends both on localized growth, which can be controlled by the plant hormone auxin, and directional growth, which is dependent on each cell's microtubule cytoskeleton. In this paper we show that at the shoot apex where organs initiate the orientation of the microtubule cytoskeleton is correlated with the orientation of the auxin transporter PIN1, suggesting coordination between growth patterning at the tissue level and directional growth at the cellular level. Recent work has indicated that mechanical signals play a role in orienting the plant microtubule network, and here we show that such signals can also orient PIN1. In addition, we demonstrate through mathematical modeling that an auxin transport system that is coordinated by mechanical signals akin to those we observed in vivo is sufficient to give rise to the patterns of organ outgrowth found in the plant *Arabidopsis thaliana*.

## Introduction

Several recent sets of observations and recent predictive models of phyllotaxis are consistent with the possibility that cells in the shoot apical meristem (SAM) can sense the auxin concentration of their nearest neighbors [1],[2]. The apparent response to high auxin levels in a neighboring cell is to direct the plasma membrane protein PIN-FORMED 1 (PIN1) to the membrane adjacent to the high-auxin neighbor, such that the PIN1 distribution around each cell can be predicted from the auxin concentration in surrounding cells. As PIN1 is an auxin efflux carrier [3], the result of this is an auxin circulatory system that responds to auxin concentration. The pattern-generating properties of this novel type of regulated developmental process, a regulated transport system, include the ability to specify the phyllotactic pattern [1],[2].

A challenge presented by these observations and associated hypotheses is that there is no known mechanism of auxin perception that could cause the coordinated localization of PIN1 in neighboring cells. The best understood mechanism for auxin perception involves the auxin-dependent activation of an SCF complex in each cell, causing the active degradation of transcription inhibitors, thereby allowing transcription of auxin-activated genes [4]. There is no directionality to such a mechanism, such that it could regulate the asymmetric distribution of PIN1 in response to external auxin signals.

Another conundrum in the study of phyllotaxis is the ability of molecules with very different properties to induce leaf or flower primordia. The successful models for phyllotaxis are based on the fact that a drop of auxin placed on a meristem causes the formation of a new leaf or flower, and therefore that a peak in auxin concentration in the meristematic peripheral zone is sufficient to activate primordium formation [1],[2]. Observations of auxin-regulated reporter genes in meristems are in accord with the idea that high auxin concentration causes primordia to form [2],[5], as are experiments in which auxin concentration is changed by mutations in biosynthetic genes [6] or by mutations or treatments that stop PIN1-dependent auxin transport [7]. However, it has also been shown that new primordia or phyllotactic disruptions can be induced by the application of substances other than auxin, such as pectin methyl esterase [8] or expansin [9]. As both of these proteins alter cell wall strength locally, their global impact on phyllotaxis must be indirect [8],[9].

We propose that both unexplained phenomena—the ability of cells to directionally respond to the auxin concentrations of their neighbors and the ability of cell-wall-altering substances to modify phyllotactic patterns—can be explained by the hypothesis that PIN1 localization depends on mechanical forces experienced by each cell in an epidermal shoot tissue under tension. As auxin induces local growth [10], perception by a cell of the expansion of its neighbor could cause the plasma membrane adjacent to the expanding neighbor to accumulate PIN1 protein. This would in turn cause the cell to export auxin in the direction of the expanding neighbor, both increasing its auxin concentration and providing positive feedback to the expansion. In this way cell wall strength and auxin concentration could be causally related in a feedback loop.

It has recently been shown that the epidermal cells of the SAM of *Arabidopsis thaliana* respond to applied stress by reorganizing their cortical microtubule arrays to be parallel to the direction of largest principal stress [11], which we define here as the axis of maximal mechanical tension. We use this assay of cellular stress and live imaging of the subcellular localization of a fluorescently tagged PIN1 protein in meristematic cells, along with a series of treatments that affect tissue stress, to show that PIN1 localization is correlated with the direction of the microtubule array in untreated SAMs, and in SAMs after a variety of treatments that change the microtubule readout of the cellular perception of mechanical stress. This indicates that PIN1 localization responds to local stress, and therefore that the subcellular localization of PIN1, and consequently the direction of auxin transport, could indeed be regulated as a response to local cell expansion.

## Results

### PIN1 Localization and Microtubule Array Orientations Are Highly Correlated in the SAM

**Figure 1:**
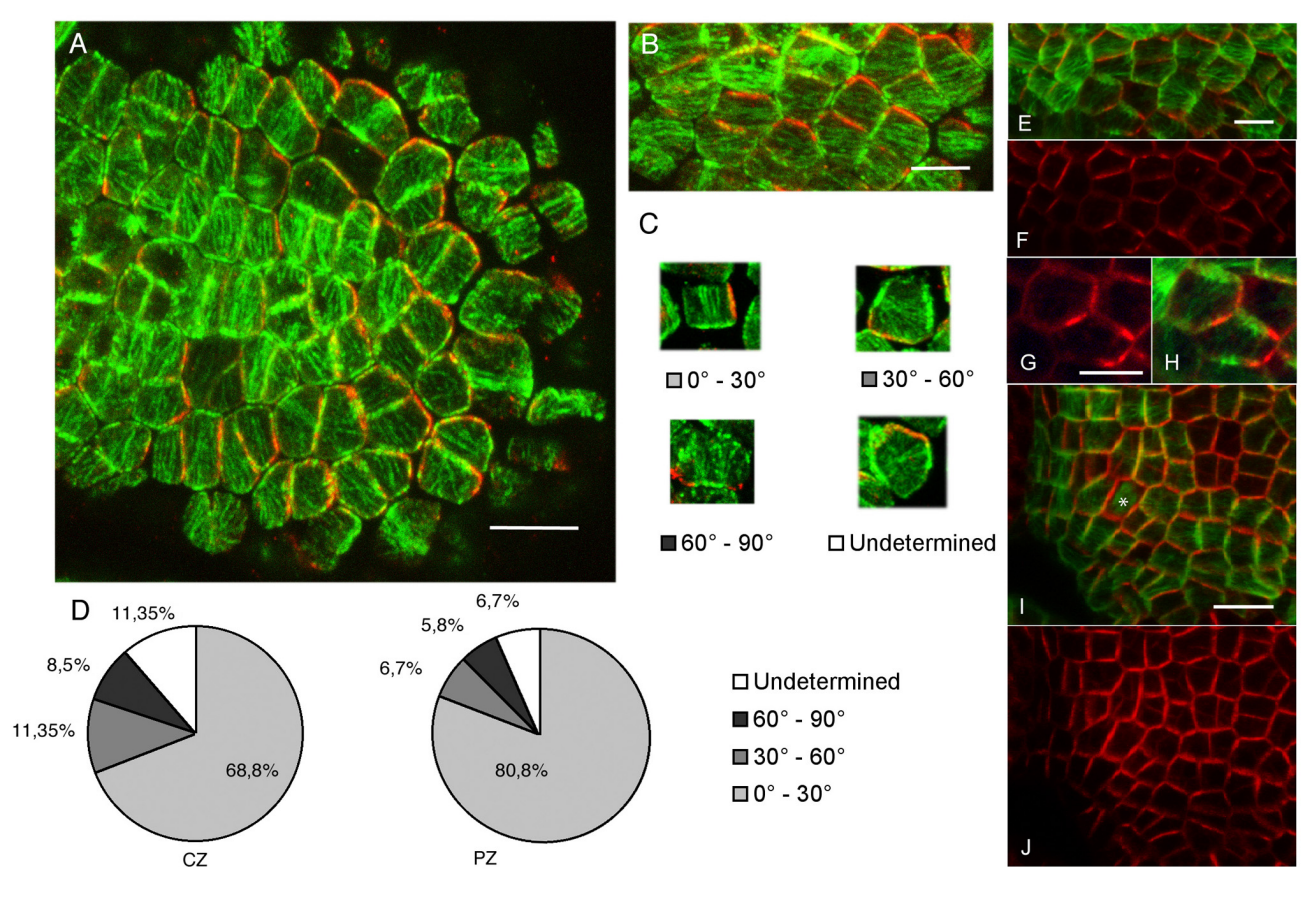
Microtubule and PIN1 orientations are correlated. (A) Immunolocalization of PIN1 (red) and α-tubulin (green) in a thick section through the surface of the meristem. Scale bar: 10 µm. (B) Close-up of the double PIN1–microtubule immunosignal in the boundary domain: PIN1 (red) and microtubule (green) patterns are correlated. Scale bar: 5 µm. (C) Examples of different degrees of correlation between microtubule bundle orientation and PIN1 localization, as quantified in (D). (D) Quantifications of the different classes of behavior in the center zone (CZ) and peripheral zone (PZ) of the meristem (*n* = 614 cells). (E–J) Correlations between PIN1 polarities and microtubule orientations similar to those seen in (B) are observed in living plants expressing PIN1-GFP (red) and TagRFP-MAP4 (green) in the boundary domain (E–H) and in incipient primordia (asterisk) (I and J). Scale bars for (E), (G), and (I): 5 µm.

We used dual immunolabeling to examine the spatial relationship between epidermal microtubule arrays and PIN1 localization. Although the degree of microtubule array anisotropy varies from cell to cell, in general we observed a good correlation between PIN1 localization and microtubule orientation, with PIN1 usually being localized towards an anticlinal wall that was parallel to the microtubules, as viewed from above. This was least obvious in the central meristem region (where microtubule orientation is known to be unstable [11]) (Figure 1A) and most obvious in the boundary regions between primordia and the meristem (Figure 1B). To analyze this in more detail we quantified the percentage of cells showing a clear correlation by measuring the angle formed between the orientation of PIN1 and the microtubule bundles (Figure 1C and 1D). For the central meristem region there was a clear correlation in the majority of those cells where assessment could be done (69%, Figure 1D). In the peripheral zone (which includes boundary regions) a greater proportion of cells showed a clear correlation (81%, Figure 1D), and this proportion rose to 100% when considering the boundary zone alone. Further correlations could also be detected when individual microtubule arrays exhibited more than one orientation, but these cases were considered not aligned overall in our classification scheme (Figure 1C and 1D). We also imaged living meristems expressing TagRFP-MAP4 and PIN1-GFP to help obtain a clear view of the correlation across the curved surface of the epidermis. In agreement with the immunolocalization data we observed clear correlations between PIN1 polarities and microtubule array orientations in boundary regions (Figure 1E–1H). In addition, we were able to assess those regions where new PIN1 convergences were forming (the future sites of new primordia) and found that here, too, PIN1 and microtubule orientations were well correlated (Figure 1I and 1J).

These results indicate that despite the dynamic behavior of both PIN1 [5] and microtubule arrays [11] in the SAM, PIN1 localization and microtubule arrays are coordinated.

### PIN1 Reorients Similarly to Microtubules in Response to Ablation

**Figure 2:**
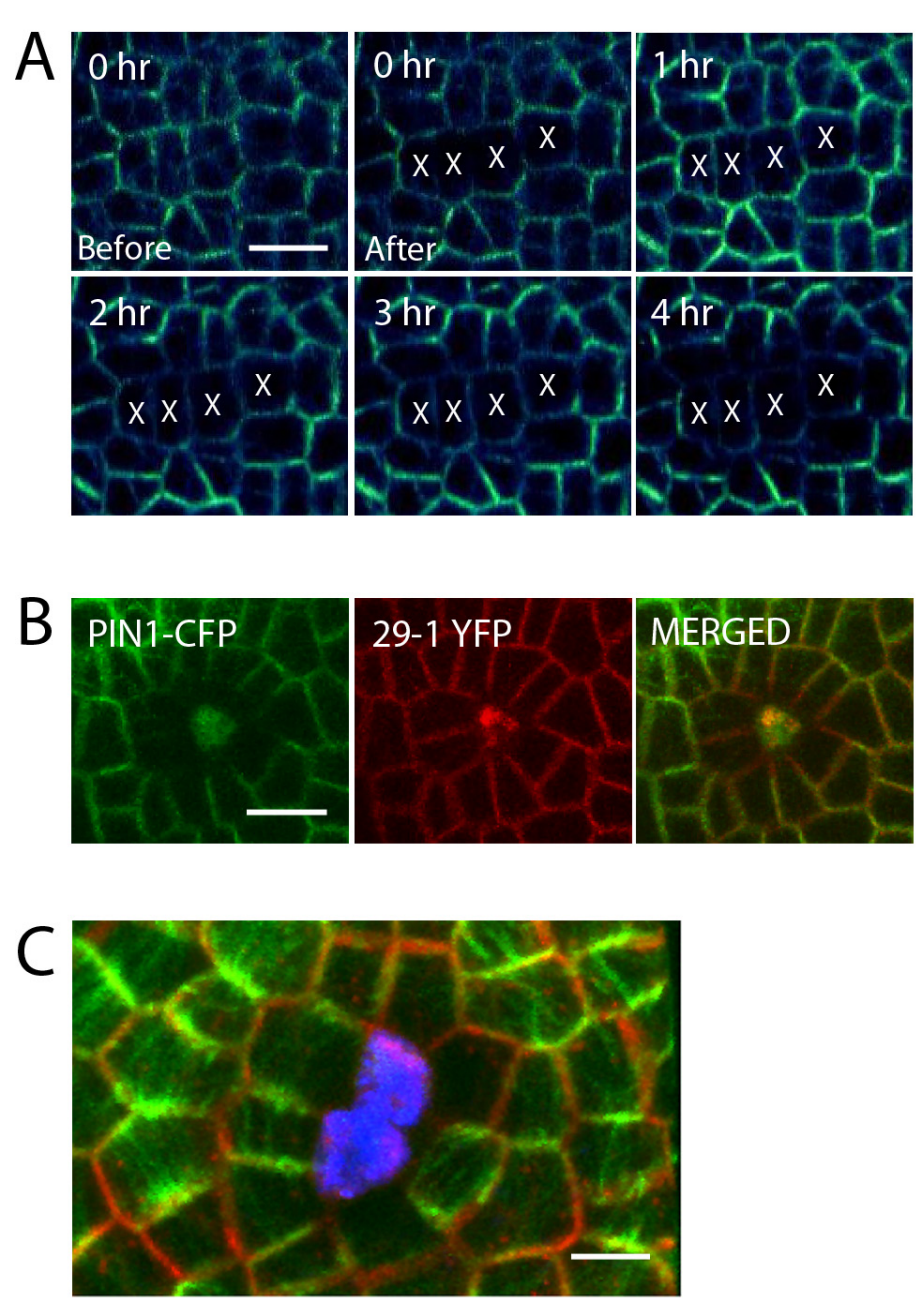
Reorientation of PIN1 polarity after ablation. Time series showing changing localization of PIN1-GFP in response to laser ablation. (A) A vertical file of cells (marked “X”) was targeted with a pulsed laser. PIN1 localization was first detected to change by 2 h after laser treatment. Scale bar: 10 µm. (B) Visualization of membrane marker 29-1 fused to YFP together with PIN1-CFP after laser ablation shows that the relocalization response is specific to PIN1. Scale bar: 10 µm. (C) Co-alignment of PIN1-GFP (red) and microtubules (green) after ablation. Ablated cells are stained with propidium iodide (blue). Scale bar: 5 µm.

Previously we demonstrated that the interphase microtubules of meristem epidermal cells located near laser-ablated cells reorient circumferentially around a wound [11]. To test whether PIN1 also reorients in response to laser ablation we conducted time-lapse imaging of PIN1-GFP-expressing cells in the meristem epidermis after laser ablation. We found that within 2 h PIN1 signal in cells adjacent to ablation sites started to shift such that over the course of several hours PIN1 became predominantly localized at the ends of cells farthest away from the nearby wound (Figure 2A). This did not appear to be a general response of membrane proteins to wounding since the membrane marker 29-1 [12] failed to show such a relocalization (Figure 2B). To directly test whether the PIN1 relocalization response resulted in a maintenance of coordination between PIN1 polarity and microtubule array orientation, we ablated cells of doubly labeled plants and found that PIN1 protein was typically localized to membrane domains that were parallel to the visible microtubule orientations, as observed for unwounded meristem tissues (Figure 2C).

### PIN1 and Microtubule Orientations Do Not Depend Directly on Each Other

**Figure 3:**
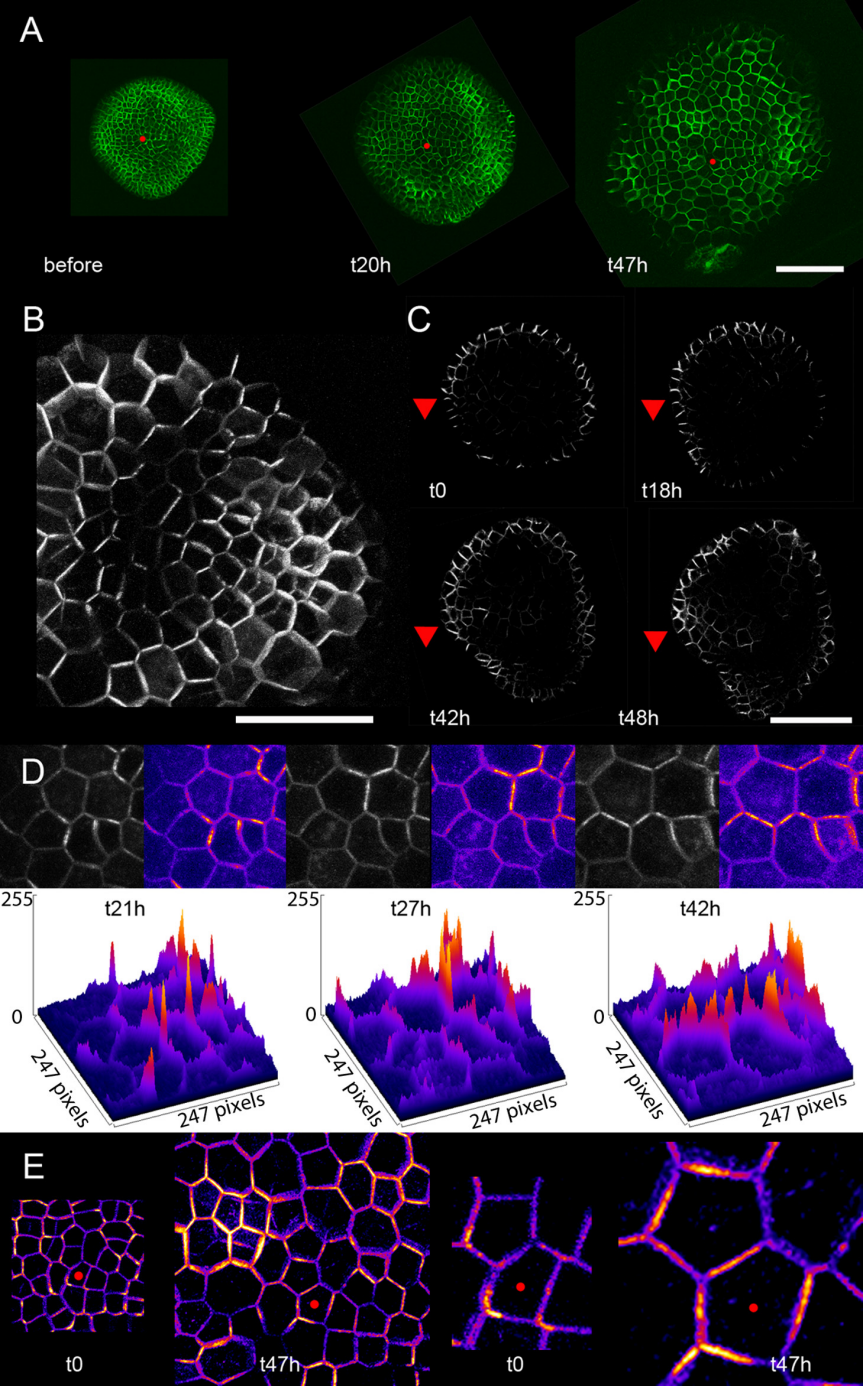
PIN1 behavior in the absence of microtubules. (A) PIN1 behavior at the meristem surface after oryzalin treatment. The absence of cell division and the enlargement of the cells are consistent with the impact of microtubule depolymerization on growth. Nevertheless, differential expression of PIN1 is maintained, and new peaks of PIN1 expression arise 20 h (middle panel) and 47 h (right panel) after microtubule depolymerization. The red dot marks the same cell at the three time points. Scale bar: 50 µm. (B) Close-up of the surface of a meristem expressing PIN1-GFP 67 h after microtubule depolymerization. The PIN1 signal is weaker in the boundary and is polarized in a divergent pattern, as observed in untreated meristems. Scale bar: 20 µm. (C) Transverse sections through a PIN1-GFP meristem treated with oryzalin. As primordia arise, the GFP signal is also detected at sites where the provasculature is initiated (arrowhead), as observed in unteated meristems. Scale bar: 50 µm. (D) Kinetics of PIN1-GFP reorientation at the surface of an oryzalin-treated meristem. The GFP signal (grayscale) is color coded (upper panels) and represented as histograms (lower panels) to better visualize the differences in GFP signals from one time point to another (from left to right 21 h, 27 h, and 42 h after oryzalin treatment). The GFP signal switches from one side of the cell to another, showing that PIN1 retains the ability to reorient in the absence of microtubules. (E) Close-ups of the surface of the same meristem expressing PIN1-GFP before and 47 h after oryzalin treatment. The PIN1-GFP signal becomes broader within the cell after long-term oryzalin treatment. The red dot marks the same cell at the two time points.

The correlations between PIN1 polarities and microtubule array orientations suggest the possibility of a direct causal connection. Although previous studies have shown that microtubules are not directly required for polar PIN1 targeting [13],[14], it has been demonstrated that the microtubule array plays an indirect role in orienting PIN1 polarity [14]. To further investigate this relationship in the SAM we examined PIN1 behavior in the SAM epidermis after depolymerization of the microtubules using oryzalin. As described previously, meristems continue to grow after oryzalin treatment, with the size of the cells also increasing due to the absence of cytokinesis [15],[16]. Oryzalin treatment of PIN1-GFP-expressing meristems revealed that differential PIN1-GFP expression and localization are maintained in the absence of microtubules. Notably, local regions of intense PIN1 signal could be identified in the epidermis and internal layers of the SAM (Figure 3A–3C). These regions corresponded to the position of new primordia (Figure3A and 3B) and concomitant provascular development (Figure 3C). The PIN1-GFP signal was not homogenously distributed on the membrane, notably in the boundary domain where PIN1 polarities localize both towards and away from the developing primordium, as observed in control meristems (Figure 3B; [5]). Lastly, when conducting time-lapse imaging of the PIN1-GFP signal, we observed that PIN1 recruitment could shift from one membrane to another several days after oryzalin treatment (Figure 3D). Altogether, these data are consistent with previous reports showing that phyllotaxis is not altered several days after oryzalin treatment [11],[15], and demonstrate that the general patterns of PIN1 localization found in the SAM are not directly dependent on microtubule orientation.

When analyzing more closely the PIN1-GFP signal in oryzalin-treated meristems, we observed that, while PIN1 maintained its ability to reorient, its distribution within a given membrane was broader after oryzalin treatment than before (Figure 3E). Notably, while the PIN1-GFP signal was often found to be concentrated at cell vertices in the presence of microtubules, the distribution of the signal became more homogeneous within a membrane after oryzalin treatment. This suggests that, while microtubules are not necessary for PIN1 reorientation, they contribute indirectly to PIN1 localization, as found previously [14].

### Microtubule and PIN1 Reorientation in Response to Wounding Is Robust to Changes in Auxin Distribution and Transport

Our results so far agree with earlier studies that PIN1 localization does not depend directly on the microtubule cytoskeleton, but our results also show that PIN1 localization and microtubule orientation are not independent. A possible explanation of the PIN1–microtubule correlation is that both microtubule orientation and PIN1 polarity are regulated by auxin gradients or transport directions. Evidence supporting this proposal comes from the observation that locally applied auxin is capable of influencing PIN1 polarity in a directional manner [17]. During this process it seems likely that microtubules would be correlated with PIN1 polarities, as they are during normal organ development. To test the dependence of microtubule and PIN1 patterning on auxin gradients and transport we examined their responses to wounding when auxin transport and gradients were disrupted.

**Figure 4:**
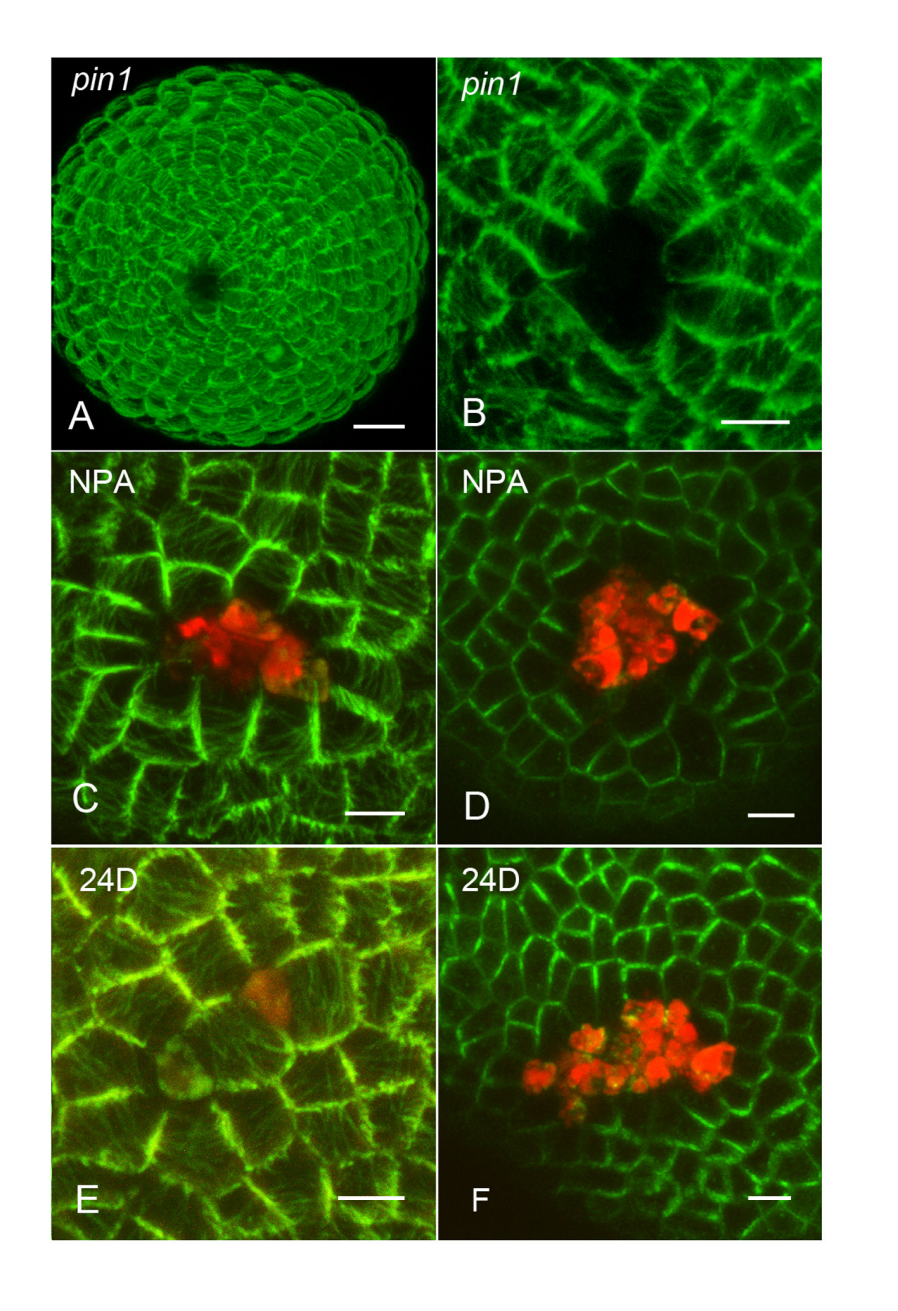
PIN1 and microtubules realign in response to laser ablation when auxin transport and distribution is altered. (A) Confocal projection showing the orientation of microtubule arrays at the *pin1* mutant meristem summit after laser ablation. (B) Close-up of cells in (A), showing circumferential microtubule orientation in cells surrounding ablation site. Cells at least one cell distant from the wound exhibit circumferential orientation. Both microtubules (C) and PIN1 (D) reorient circumferentially around wounds 24 h after NPA treatment. Note that in (D) PIN1 is oriented away from the wound in cells several cells distant from the wound. Similar behavior is observed for microtubules (E) and PIN1 (F) after 2,4-D treatment. Dead cells are marked by propidium iodide staining (red). Note that two cells separated by an intervening cell are ablated in E. Scale bar for (A): 10 µm. Scale bars for (B–F): 5 µm.

First we conducted ablation experiments in the *pin1* mutant background and observed the microtubule response. Untreated *pin1* meristems exhibited microtubule orientations similar to wild-type, with a circumferential pattern around the lower meristem flanks and a more random pattern at the tip (data not shown). Likewise, we found that microtubules responded to ablation as they do in wild-type by becoming circumferentially oriented around the ablation site (Figure 4A and 4B). Next we treated apices with N-1-naphthylphthalamic acid (NPA), an auxin transport inhibitor, and examined both the PIN1 and microtubule response to ablation. Again, we found that in both cases the ablation response was the same as for wild-type untreated meristems (Figure 4C and 4D). Even several cell diameters from the ablated cell, PIN1 became oriented away from the ablation site, demonstrating that auxin transport is not required for this ablation-induced orientation signal (Figure 4D). Despite disruptions to auxin transport, differences in auxin concentration between cells may still conceivably be playing an instructive role during these experiments. To try to disrupt any such gradients we next applied 2,4-dichlorophenoxyacetic acid (2,4-D) to the meristem, an auxin analog that freely diffuses between cells, for 24 h before ablating and observing the PIN1 and microtubule response. Uniform PIN1 expression was observed after 24 h, demonstrating that 2,4-D effectively penetrated the tissue [5]. Nevertheless, as for untreated meristems, both PIN1 and the microtubule arrays of cells surrounding the ablated cell reoriented to point away from or form concentric patterns around the wound, respectively (Figure 4E and 4F). Again, at least in the case of PIN1, orientation away from the ablation site was observed at a distance (Figure 4F).

These data show that although PIN1 polarities are sensitive to local auxin application [17], coordinated directional changes in PIN1 polarity can also occur when auxin distribution and transport are disrupted.

### Isoxaben Induces Hyperalignment of Both PIN1 and Microtubules to Predicted Stress Directions

Previously we demonstrated that microtubule orientation can be influenced by mechanical stress and that microtubule orientations in the meristem epidermis align along the predicted maximal principal stress directions [11]. To further assess the response of microtubules to stress and investigate whether PIN1 also responds to stress we attempted to alter stress levels by treating meristem cells with isoxaben, while observing the microtubule and PIN1 response. Isoxaben is a well-documented inhibitor of cellulose synthesis that likely interacts with the cellulose synthases CESA3 and CESA6 and induces the internalization and sequestration of CESA complexes in small vesicular bodies [18]–[20]. As the thickness of the wall decreases in growing isoxaben-treated cells, the resistance of the wall to the internal turgor pressure will decrease, which also means that mechanical stress (force per cross-section area) in the wall is expected to increase. We note that the documented short-term effects of isoxaben and 2,6-dichlorobenzonitrile, another cellulose synthesis inhibitor, on microtubule orientation remains unclear since both have been shown to induce either randomization of microtubule orientation [21],[22] or microtubule reorientation [23] after a few hours.

**Figure 5:**
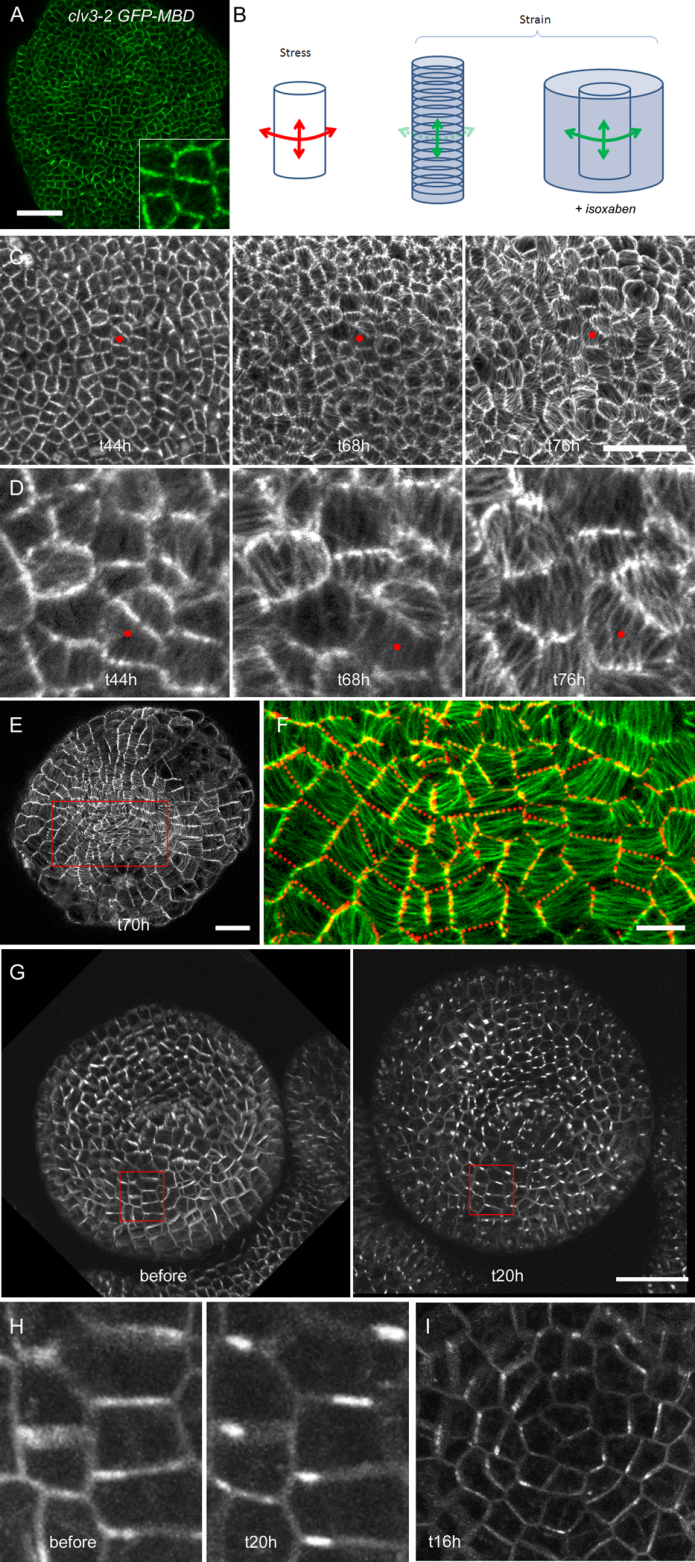
Microtubule and PIN1 behavior when cellulose synthesis is inhibited with isoxaben. (A) Surface of a *clv3-2* GFP-MBD meristem before isoxaben treatment, with magnified insert (later time points after treatment shown in [C] and [D]). Note the presence of strong GFP signal on all sides for many of the cells, indicating a random alignment of microtubules in those cells. Scale bar: 20 µm. (B) Theoretical impact of isoxaben on patterns of stress (red) and strain (green) in a cylindrical pressure vessel. The main direction of stress in a cylindrical pressure vessel is circumferential. As plant cells reinforce their walls parallel to the main stress, the residual axial stress drives a strain perpendicular to the main stress. If cellulose deposition is inhibited, the strain follows the stress pattern, i.e., the circumferential strain becomes higher than the axial strain. (C) Impact of isoxaben on microtubule behavior in the *clv3-2* GFP-MBD line at different time points after application (microtubule arrangements before application are shown in [A]). Cell growth continues, and microtubules gradually form thick bundles. There is no apparent coordination in the orientations. The red dot marks the same cell at the three time points. Scale bar: 40 µm. (D) Close-ups from (C) showing microtubule orientations in relation to the cell shapes after isoxaben treatment. (E) Surface of a GFP-MBD meristem 70 h after the isoxaben treatment. Note the presence of circumferential bundles of microtubules. Scale bar: 20 µm. (F) Close-up from (E) showing that the microtubules become circumferential even at the tip of the meristem, and this can be correlated to the dome shape of the meristem in the wild-type background. The red dotted lines represent the position of the anticlinal walls (reconstructed from sections through the stack). Scale bar: 10 µm. (G) Surface of a meristem expressing PIN1-GFP before and 20 h after isoxaben treatment. The GFP signal becomes localized to a subdomain of the plasma membrane. Scale bar: 30 µm. (H) Close-ups from (G): the GFP signal is concentrated on the circumferential membrane near a vertex. (I) Surface of a meristem expressing PIN1-GFP 16 h after isoxaben treatment, showing a preferential localization of PIN1 on the circumferential membranes.

First we analyzed microtubule behavior in the central zones of *clv3-2* meristems (Figure 5A), which, being roughly flat, we presume exhibit more-or-less isotropic stress patterns. We first grew the *clv3-2* GFP-MBD plants on NPA to prevent organ formation and differential growth at the apex and then immersed the seedlings in 20 µM isoxaben for 20 h on day 1 and day 2. Before isoxaben treatment, most of the cells in the *clv3-2* GFP-MBD background displayed random microtubule orientations (Figure 5A), as is also seen in the central zone of wild-type plants [11]. After isoxaben treatment, despite its effect on wall synthesis, growth continued and cell size increased dramatically as cytokinesis did not occur (Figure 5C). Time-lapse analysis of microtubule behavior in the *clv3-2* GFP-MBD meristems showed that the random patterns of microtubules initially observed stabilized into highly bundled arrays with clear orientations. At the same time, cell expansion occurred to a large extent parallel to the observed microtubule orientations, in contrast to the usual growth behavior of cells during normal development [24] (Figure 5D; Video S1). This observation is consistent with the proposal that by inhibiting cellulose synthesis, isoxaben prevents the microfibrils from aligning parallel to the largest principal stresses, thus enabling significant growth parallel to these stresses (Figure 5B). Next we investigated the impact of isoxaben on microtubules in wild-type apices using the GFP-MBD line. In contrast to the *clv3-2* meristems, which display a flat surface at the apex, the hemispherical shape of the GFP-MBD meristems is expected to generate a supracellular pattern of stress that is circumferential at the base of the meristem and isotropic only at the very tip of the meristem [11]. As observed in the *clv3-2* background, we observed the formation of microtubule bundles in every cell of the meristem surface (Figure 5E). Furthermore, almost every cell displayed a microtubule orientation that followed the expected supracellular stress pattern, even near the very top of the meristem (Figure 5F), in contrast to control meristems, where microtubules aligned circumferentially farther from the center [11],[25],[26].

Next we investigated how PIN1 responds to isoxaben treatment. The PIN1-GFP line was grown in the presence of NPA and then immersed in 20 µM isoxaben for 20 h. After isoxaben treatment, the PIN1 signal exhibited three main features. First, the signal became extremely bright at the plasma membrane, with no signal in internal vesicles, showing that PIN1 internalization was abolished (Figure 5G and 5H). Second, the PIN1 signal became almost exclusively localized to subdomains of the membrane, usually with a stronger signal near one vertex (Figure 5H). Finally, we observed that the new isoxaben-induced PIN1 pattern became circumferential, i.e., parallel to the predicted stress directions and microtubule orientations, consistent with a model in which PIN1 orientation depends on mechanical stress (Figure 5I). Equivalent experiments with a control GFP-LTI6b membrane marker indicated no effect of the isoxaben treatment on GFP-LTI6b localization (data not shown).

These data show that a presumed increase in wall stress due to the inhibition of cellulose synthesis correlates with a more ordered and oriented pattern for both microtubules and PIN1 that matches predicted stress patterns, supporting the conclusion that both microtubule orientation and PIN1 localization are regulated by stress.

### PID Is Required for Coupling PIN1 Polarities to Microtubule Orientations in Response to Wounding

**Figure 6:**
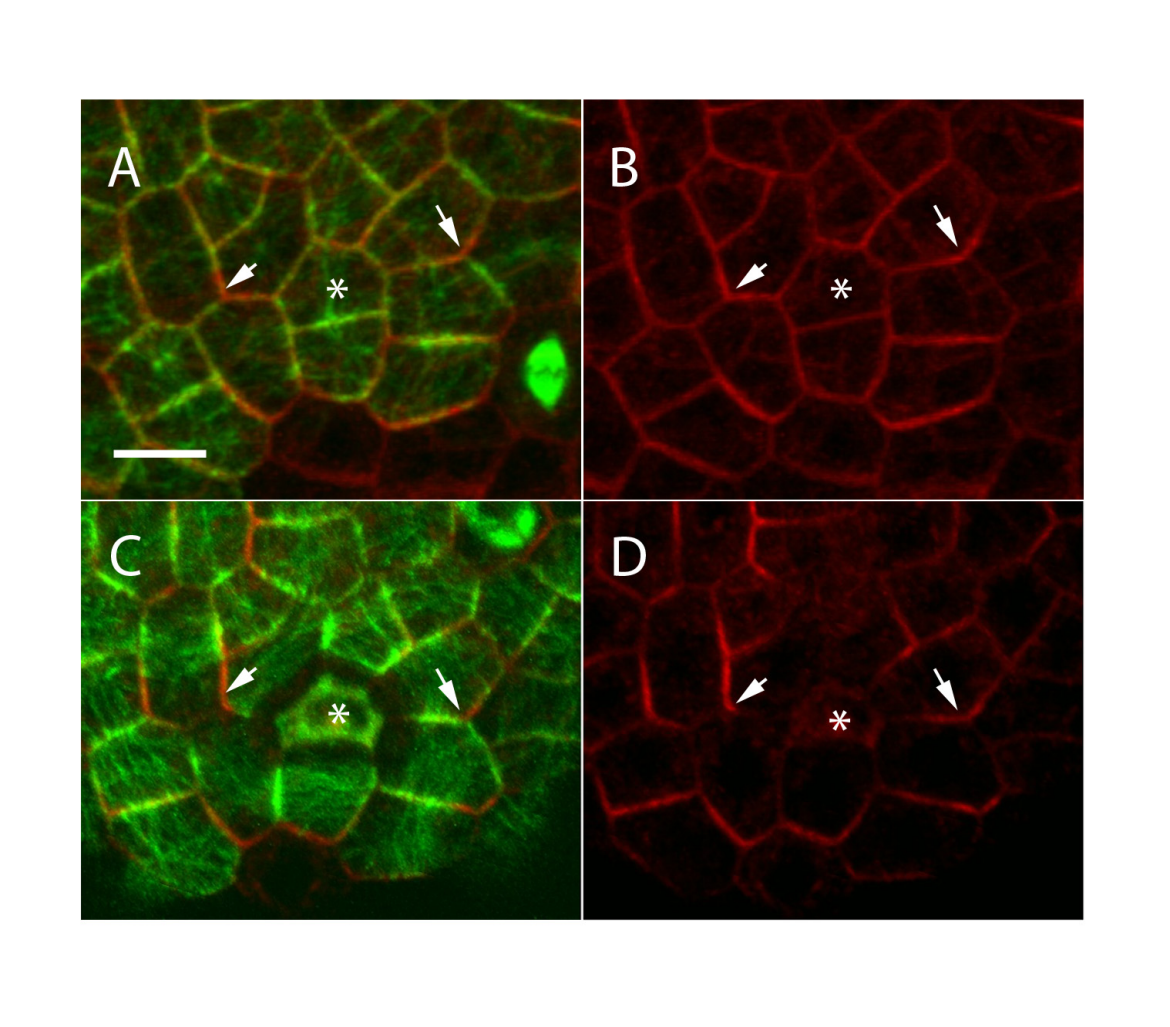
PID is required for relocalization of PIN1 but not microtubules after ablation. Visualization of PIN1-GFP (red) and TagRFP-MAP4 (green) before (A and B) and after (C and D) laser-induced cell ablation (asterisk) in a *pid* mutant background. After ablation microtubules reorient around the wound similarly to wild-type. In contrast, PIN1 repolarization in the *pid* mutant is significantly reduced and remains uncorrelated with microtubule orientations (arrows). Scale bar: 5 µm.

Given the tight coupling between PIN1 polarity and microtubule orientation we sought to test whether PINOID (PID), a known regulator of PIN1 polarity, might also regulate microtubule orientation. In the *pid* mutant apex, PIN1 was basally localized, as shown previously [27]. This localization correlated with microtubule orientation around the meristem flanks since in these regions the microtubule orientation was circumferential. At the tip the correlation was not as obvious, as in wild-type. To investigate further we used laser ablation on doubly labeled plants and assessed the reorientation response. We found that while the microtubules reoriented as in wild-type, the PIN1 response was significantly reduced, with PIN1 polarity shifting only minimally in the surrounding cells (Figure 6). These data show not only that PID is required for mediating an apical or basal PIN1 orientation, but also that PID is required for reorientation of PIN1 in a more general sense and that microtubule orientations do not directly depend on PID.

### Mechanical Stresses Can Pattern Phyllotaxis by Regulating Auxin Transport

**Figure 7:**
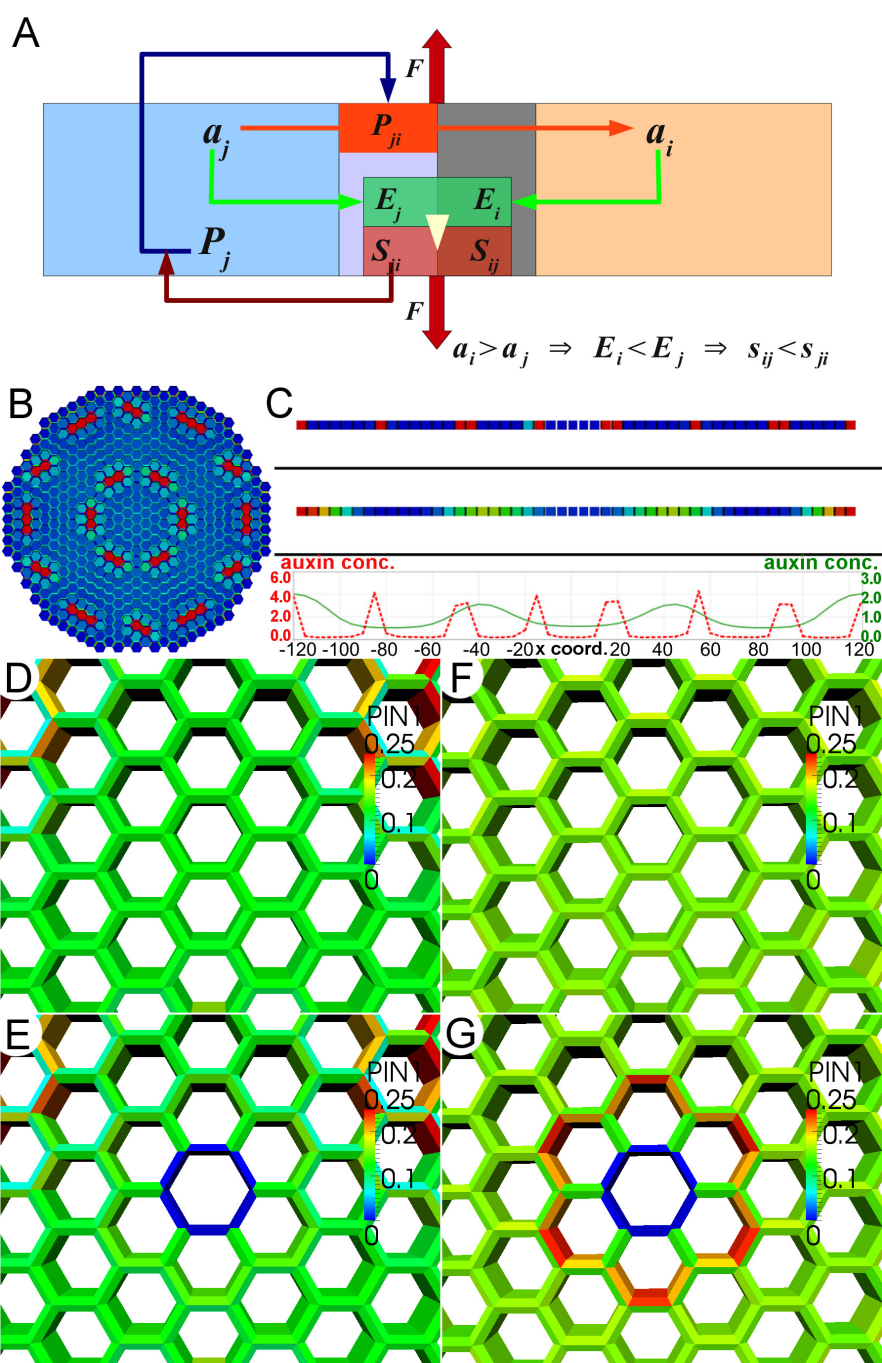
Mathematical model of auxin transport and mechanical stress. (A) Schematic representation of the interactions leading to a pattern-forming behavior in the model. Auxin (*a*) is transported out of cell *j* to cell *i* by the PIN1 (*P*) proteins localized to the membrane, *P_ji_*. Auxin concentration in each cell affects the elasticity (*E_i_* and *E_j_*) of the adjacent wall, which influences the mechanical stress (*S_ij_* and *S_ji_*) perceived in both parts of the wall between the cells as a result of the force *F*. For example *a_i_*>*a_j_* leads to *E_i_*<*E_j_*, which in turn causes *S_ij_*<*S_ji_*. The cycling of PIN1 between cytosol, *P_j_*, and the membrane, *P_ji_*, depends on these stresses, and larger *S_ij_* causes stronger allocation of *P_j_* to *P_ji_*. (B) Example of the auxin pattern created spontaneously in the model by applying uniform tension to a two-dimensional template. (C) Spacing of emerging auxin peaks can be controlled by adjustment of model parameters. Patterns of auxin distribution obtained in two different one-dimensional simulations of the model show different arrangements of auxin peaks (compare red versus green peak profiles). (D–G) Comparison of the behavior of the new model with a previously proposed auxin transport model [1] in the case of the response to ablation of a cell. In the auxin-concentration-based model, removal of the cell in the region of uniform auxin distribution (D) causes only minor response of the PIN1 polarization in the cells neighboring the removed cell (E). In the stress-based model, we observed much stronger, outward PIN1 polarization in the cells closest to the ablated region (G), as compared to the situation prior to ablation (F). The ablation results for the new model provide a better fit to the experimental data (see Figures 2 and 4).

Previously we used mathematical modeling to show that mechanical stress patterns predict the patterns of microtubules. So far our data suggest a role for mechanical signals in regulating not only microtubules but also PIN1. To test whether mechanical signals are sufficient to explain the observed patterns of PIN1 localization associated with cell ablations and with formation of the phyllotactic pattern, we investigated the hypothesis that PIN1 in each cell localizes towards the walls that are most mechanically stressed using computer modeling (Text S1). For this purpose we treat the cell wall surrounding a cell as a distinct mechanical compartment and compute stresses separately in each adjacent cell wall. Thus, we postulate the existence of stress-induced signals from the cell wall that act only locally to promote accumulation of PIN1 at the nearest membrane (Figure 7A; Text S1). The model also assumes that auxin-induced cell wall loosening in response to auxin concentrations inside a cell is limited to the wall compartments belonging to that cell. The mechanical part was implemented using a finite element method (FEM) description and auxin-induced growth by weakening of the wall rigidity, as described previously [11]. For auxin transport we used a description following the chemiosmotic transport theory, with parameter values from experimental estimates [1],[28],[29]. We assumed symmetric localization of influx carriers and used equilibrium values for transport between cytosol and wall compartments to get a cell-based description [30]. Note that this implicit description of auxin in the walls has been shown not to alter behavior of the auxin transport model [1]. The difference from previous models is that PIN1 dynamics is now driven by wall stresses rather than auxin concentrations in neighboring cells (Figure 7A; see [1]). Hence, the model mechanisms are now based on mechanical and chemical interactions within single cells or between neighboring cell wall compartments and are not dependent on chemical signals between cells. Since we assume PIN1 cycling dynamics to be in quasi-equilibrium, PIN1 localization can be interpreted as being the result of wall stresses either inducing PIN1 exocytosis or reducing endocytosis. The model behavior also depends on two additional assumptions. First, since PIN1 is localized mainly in anticlinal—and not periclinal—walls in the shoot epidermis [5],[31], we assumed that PIN1 does not localize towards walls where there are no neighboring cells on the other side. This assumption is also supported by the observation made in cell suspension cultures that PIN1 is only present in membranes that are adjacent to neighboring cells [14]. Second, previous analysis has shown that, if the two adjacent cell walls are treated as a single compartment, a dependence of PIN1 cycling on wall signals does not lead to a pattern-forming mechanism [30],[31] since a strong wall signal would lead to increased PIN1 on both sides of a wall, which is not detected in the epidermis [5],[31]. Hence, we included in the model separate compartments for both wall segments between two cells, where the two wall compartments may have different mechanical properties (Figure 7A; Text S1).

A spacing mechanism for primordia positioning together with tissue growth and a central zone unable to produce organs is, in theory, sufficient to produce phyllotactic patterns of various symmetries [2],[32],[33]. To test the model's capability of generating patterns, we simulated the model on a two-dimensional tissue representing shoot epidermal tissue. When the tissue was under tension it generated a periodic pattern of auxin distribution from a homogeneous state (Figure 7A). To further investigate the model's capability of generating patterns we did one-dimensional simulations together with linear stability analysis of the homogeneous fixed point. The analysis showed an initial wavelength-dependent dynamics from the homogeneous state, and the simulations resulted in a peaked pattern with a similar parameter-dependent wavelength (Figure 7C; Text S1). Taken together, these results show that a mechanism that distributes PIN1 localization according to cell wall stress is capable of generating phyllotactic-like patterns and that the behavior of such a model is similar to that of an earlier proposed model in which PIN1 distribution patterns were governed by relative auxin concentration in neighboring cells [1].

Although the auxin-concentration-based and the stress-based models for PIN1 dynamics in many cases create very similar results, there are differences since tissue geometry and growth feed back to the tissue stresses. For example, in the valley between the shoot and a primordium, stresses are along the valley and amplify a tendency of PIN1 to point towards or away from primordia, as seen in experiments [5],[11]. Also, at the plant stem a stress-based model correctly predicts the apical basal preference for PIN1 localization [27]. One way to discern between molecular-based and stress-based models would be to simulate a situation in which mechanical stresses are perturbed. We did this by following PIN1 dynamics in simulations of the two models in the case of laser-induced cell ablations (Figure 7D–7G). Such ablations induce circumferential stresses in the cells surrounding the ablated cell [11], and it is clear that only the stress-based model captures the strong reorganization of PIN1 away from the ablated cell that is seen in the experiments (see Figures 2 and 4). In conclusion, the model shows that a stress-based mechanism can produce the phyllotactic patterns observed experimentally, and the predictions of models based on different tissue geometries and on ablation experiments favor a mechanical stress signal for orienting PIN1 in the shoot epidermis.

## Discussion

Although microtubule orientations and PIN protein localizations are known to mark a common apical–basal axis in the root [14], our findings in the SAM show an unexpected level of coordination between what are, in the SAM, highly dynamic subcellular markers. In the SAM, PIN1 polarities change on a time course of hours, with reversals in polarity associated with primordium formation [5]. Microtubules also exhibit dynamic reorientations, especially at the meristem tip [11]. That these two dynamic cellular components are highly coordinated suggests either that they are causally dependent on one another or that their localizations are both regulated by a common upstream factor. Our data strongly suggests the latter since microtubule arrangements do not depend on auxin transport for their correlated response to cell ablation even at a distance from the ablation site. Also, after microtubule depolymerization, PIN1 localization remains polarized and can shift both during primordium development and in response to ablation. If auxin gradients or flux patterns are not required for coordinating PIN1 polarities and microtubule orientations, what could act as the upstream patterning agent? Considering recent findings, it seems likely that mechanical signals coordinate their activities. For example local up-regulation of a pectin methyl esterase is sufficient to induce ectopically the full program of flower development, suggesting that changes to the mechanical properties of cell walls are sufficient to induce the usual changes to both microtubule orientations as well as PIN1 polarities [8]. Also, mechanical manipulation of *Arabidopsis* roots is sufficient to induce lateral root initiation, and the earliest event so far identified marking lateral root initiation is the relocalization of PIN1 protein in root protoxylem cells [34]. We investigated the role of mechanical signals by inhibiting cellulose synthesis using isoxaben to alter cell wall properties. We reasoned that if the addition of load-bearing cellulose to growing cell walls was prevented, the stress levels for existing wall components should increase. Consistent with this proposal we found that isoxaben treatment induces hyperlocalization of PIN1 and an enhanced and stabilized supracellular pattern of microtubule array orientations [11],[21],[35]. Also we found that under these circumstances PIN1 is predominantly localized to cell corners, consistent with the fact that corners and junctions are generally associated with high stresses in mechanical structures [36]. Lastly, we note that isoxaben may cause distinct responses in different tissues since short-term isoxaben treatment of roots weakens rather than strengthens preexisting microtubule array alignments [23].

To investigate whether mechanical signals could be responsible for generating the observed patterns of PIN1 localization, we constructed a computer model that localizes PIN1 to membranes adjacent to cell walls that exhibit the highest stress. Such a model behaves similarly to previously proposed models for phyllotaxis based on differential auxin concentrations because we assume that higher auxin concentrations induce greater levels of cell wall relaxation in local cell walls compared to the cell walls of adjacent cells. Loosening of one cell wall thereby induces higher stress in the adjoining cell wall, and PIN1 becomes localized towards the cell with the most relaxed cell wall (highest auxin). Although similar to the previous chemically based models, this model is more general because wall stress depends not only on auxin but also on tissue morphology, mechanical perturbations, and the activity of any genes that modulate growth. Hence the model may explain a variety of observations in the literature that include both auxin-induced [17], mechanically induced [34], and wall-enzyme-induced changes to growth and cell polarity [8]. Lastly it is worth pointing out that a possible link between mechanical stress and auxin-based patterning of phyllotaxis has been proposed previously [37]. In this study it was found that coupling auxin distribution patterns to stress patterns could, under certain conditions, produce a reinforcement of the phyllotactic pattern. It will be of interest to explore such models further, in particular by enabling stress to regulate not only auxin transport patterns, but also local patterns of mechanical anisotropy, as suggested by the current study. Experimentally, it will be important to test whether cell walls play a role in locally regulating PIN1 membrane accumulation.

Independent of the particular mechanisms by which PIN1 and microtubule array orientations are regulated, their tight coupling in the SAM epidermis implies high-level coordination between growth direction, as patterned by microtubule arrays, and growth localization and gene expression, as patterned by the distribution of auxin, both during normal development and in response to wounding. An important future task will be to determine how universal this coordination is. Another will be to further investigate the role of mechanical signals in cell–cell communication in development and in coordinating growth and cell wall reinforcement with each other, and with stress.

## Materials and Methods

### Plant Material and Growth Conditions

The GFP-MBD line was a kind gift from Martine Pastuglia. Plants were grown and prepared for imaging as previously described [12],[15].

### Microscopy and Chemical Treatments

Microscopy was conducted as described previously [11] except that a 543-nm laser line was used to excite TagRFP-MAP4 in conjunction with 488-nm excitation of PIN1-GFP using line-by-line multitracking on a Zeiss LSM 510. Treatments with NPA were carried out as previously described [5],[11]. For 2,4-D treatment, 100 µM 2,4-D (Sigma) was diluted from a 0.1 M stock solution in DMSO. For oryzalin and isoxaben treatments whole plantlets were transferred in boxes containing solid medium without NPA, and attached by adding a lukewarm gel agarose at 0.5%. To depolymerize the microtubules, the plantlets were immersed in an aqueous solution containing oryzalin at 20 µg/ml for 3 h, then washed in water twice for 15 min on day 1. The same treatment was applied on day 2, and images of the meristem were obtained from day 1 to day 3. To inhibit cellulose deposition, the plantlets were immersed in an aqueous solution containing isoxaben at 20 µM for 20 h, then washed in water twice for 15 min on day 1. Images of the meristem were obtained from day 1 to day 3. When using the *clv3-2* background, the isoxaben treatment was repeated on day 2, and images of the meristem were obtained from day 2 to day 4. Projections of the meristem surface were generated using the Merryproj software [38], and close-ups of the same set of cells were aligned with the AlignSlice plug-in from ImageJ. All experiments were repeated at least three times, with comparable results.

### Transgenic Reporter Constructs

A photostable TagRFP-T variant [39] was modified from pTagRFP-N (Evrogen) using PCR amplification with primers Tag-RFP-Tf, 5′ ggatccATGGTGTCTAAGGGCGAAGAG 3′, and Tag-RFP-Tr, 5′ agatcttgccgcggcCTTGTACAGCTCGTCCATG 3′. Mouse MAP4 microtubule binding domain cDNA [11] was PCR-amplified with the primers MAP4f, 5′ GGATCCCAAGAAGAAGCAAAGGCTGCTGTAGGTGT 3′, and MAP4r, 5′ AGATCTTTAGCCAACGTTATCAAGTGATCCCACTTTGG 3′. A TagRFP-T-MAP4 translational fusion was cloned into the pOp6/LhGR two-component system [40] for dexamethasone-inducible misexpression of TagRFP-T-MAP4. We used a ML1p::LhGR driver containing 3.4 kb of the L1-specific ML1 gene (At4g21750) fused to the chimeric LhGR transcription factor and a 6XOp::TagRFP-T-MAP4 expression construct in a sulfadiazine-resistant T-DNA vector (ML1>>TagRFP-T-MAP4).

### Immunolocalizations

Apical inflorescences were fixed in fresh FAA solution (3.7% formaldehyde, 50% EtOH, and 5% acetic acid) under vacuum, embedded in low-melting-point wax (Aldrich), and processed for immunofluorescence. After rehydration, 6-µm sections were pretreated 1 h with 2% BSA in PBS and incubated overnight with the AP20 anti-PIN1 antiserum (Santa Cruz Biotechnology) and the monoclonal anti-α-tubulin antiserum (Sigma) respectively diluted 1∶500 and 1∶1,000 in PBS containing 0.1% BSA. After three washes in PBS with 0.1% (v/v) Tween 20, sections were incubated for 1 h with the secondary antibodies Alexa-Fluor-488-labeled donkey anti-goat and Alexa-Fluor-555-labeled donkey anti-mouse IgG (Invitrogen) diluted 1∶1,000 in PBS supplemented with 0.1% (w/v) BSA. After additional rinses in PBS plus 0.1% Tween 20, sections were mounted in Citifluor under cover slips and examined using a confocal laser scanning microscope.

## Supporting Information

Text S1 **Biomechanical model details and stability analysis.**
(0.09 MB PDF)

Video S1 **Impact of isoxaben on microtubule behavior in the** ***clv3-2*** **GFP-MBD line.** Video shows expansion of cells labeled with MAP4-GFP to show microtubule orientations. Images were obtained at 20, 44, and 76 h after isoxaben treatment. Note the directional (vertical) cell elongation on the left side of the frame and the concomitant alignment of microtubule bundles in the same orientation.
(0.49 MB MOV)

## Abbreviations

2,4-D: 2,4-dichlorophenoxyacetic acid
NPA: N-1-naphthylphthalamic acid
SAM: shoot apical meristem

## References

[1] Jonsson H, Heisler M. G, Shapiro B. E, Mjolsness E, Meyerowitz E. M (2006). An auxin-driven polarized transport model for phyllotaxis.. Proc Natl Acad Sci U S A.

[2] Smith R. S, Guyomarc'h S, Mandel T, Reinhardt D, Kuhlemeier C (2006). A plausible model of phyllotaxis.. Proc Natl Acad Sci U S A.

[3] Petrasek J, Mravec J, Bouchard R, Blakeslee J. J, Abas M (2006). PIN proteins perform a rate-limiting function in cellular auxin efflux.. Science.

[4] Chapman E. J, Estelle M (2009). Mechanism of auxin-regulated gene expression in plants.. Annu Rev Genet.

[5] Heisler M. G, Ohno C, Das P, Sieber P, Reddy G. V (2005). Patterns of auxin transport and gene expression during primordium development revealed by live imaging of the Arabidopsis inflorescence meristem.. Curr Biol.

[6] Cheng Y, Dai X, Zhao Y (2006). Auxin biosynthesis by the YUCCA flavin monooxygenases controls the formation of floral organs and vascular tissues in Arabidopsis.. Genes Dev.

[7] Okada K, Ueda J, Komaki M. K, Bell C. J, Shimura Y (1991). Requirement of the auxin polar transport system in early stages of Arabidopsis floral bud formation.. Plant Cell.

[8] Peaucelle A, Louvet R, Johansen J. N, Höfte H, Laufs P (2008). Arabidopsis phyllotaxis is controlled by the methyl-esterification status of cell-wall pectins.. Curr Biol.

[9] Fleming A. J, McQueen-Mason S, Mandel T, Kuhlemeier C (1997). Induction of leaf primordia by the cell wall protein expansin.. Science.

[10] Reinhardt D, Mandel T, Kuhlemeier C (2000). Auxin regulates the initiation and radial position of plant lateral organs.. Plant Cell.

[11] Hamant O, Heisler M. G, Jonsson H, Krupinski P, Uyttewaal M (2008). Developmental patterning by mechanical signals in Arabidopsis.. Science.

[12] Reddy G. V, Heisler M. G, Ehrhardt D. W, Meyerowitz E. M (2004). Real-time lineage analysis reveals oriented cell divisions associated with morphogenesis at the shoot apex of Arabidopsis thaliana.. Development.

[13] Geldner N, Friml J, Stierhof Y. D, Jurgens G, Palme K (2001). Auxin transport inhibitors block PIN1 cycling and vesicle trafficking.. Nature.

[14] Boutte Y, Crosnier M. T, Carraro N, Traas J, Satiat-Jeunemaitre B (2006). The plasma membrane recycling pathway and cell polarity in plants: studies on PIN proteins.. J Cell Sci.

[15] Grandjean O, Vernoux T, Laufs P, Belcram K, Mizukami Y (2004). In vivo analysis of cell division, cell growth, and differentiation at the shoot apical meristem in Arabidopsis.. Plant Cell.

[16] Corson F, Hamant O, Bohn S, Traas J, Boudaoud A (2009). Turning a plant tissue into a living cell froth through isotropic growth.. Proc Natl Acad Sci U S A.

[17] Bayer E. M, Smith R. S, Mandel T, Nakayama N, Sauer M (2009). Integration of transport-based models for phyllotaxis and midvein formation.. Genes Dev.

[18] Scheible W. R, Eshed R, Richmond T, Delmer D, Somerville C (2001). Modifications of cellulose synthase confer resistance to isoxaben and thiazolidinone herbicides in Arabidopsis Ixr1 mutants.. Proc Natl Acad Sci U S A.

[19] Paredez A. R, Somerville C. R, Ehrhardt D. W (2006). Visualization of cellulose synthase demonstrates functional association with microtubules.. Science.

[20] Gutierrez R, Lindeboom J. J, Paredez A. R, Emons A. M, Ehrhardt D. W (2009). Arabidopsis cortical microtubules position cellulose synthase delivery to the plasma membrane and interact with cellulose synthase trafficking compartments.. Nat Cell Biol.

[21] Fisher D. D, Cyr R. J (1998). Extending the microtubule/microfibril paradigm—cellulose synthesis is required for normal cortical microtubule alignment in elongating cells.. Plant Physiol.

[22] Himmelspach R, Williamson R. E, Wasteneys G. O (2003). Cellulose microfibril alignment recovers from DCB-induced disruption despite microtubule disorganization.. Plant J.

[23] Paredez A. R, Persson S, Ehrhardt D. W, Somerville C. R (2008). Genetic evidence that cellulose synthase activity influences microtubule cortical array organization.. Plant Physiol.

[24] Lloyd C, Chan J (2004). Microtubules and the shape of plants to come.. Nat Rev Mol Cell Biol.

[25] Sakaguchi S, Hogetsu T, Hara N (1988). Arrangement of cortical microtubules in the shoot apex of Vinca major L.. Planta.

[26] Marc J, Hackett W. P (1989). A new method for immunofluorescent localization of microtubules in surface cell layers—application to the shoot apical meristem of Hedera.. Protoplasma.

[27] Friml J, Yang X, Michniewicz M, Weijers D, Quint A (2004). A PINOID-dependent binary switch in apical-basal PIN polar targeting directs auxin efflux.. Science.

[28] Rubery P. H, Sheldrake A. R (1974). Carrier-mediated auxin transport.. Planta.

[29] Raven J. A (1975). Transport of indoleacetic acid in plant cells in relation to pH and electrical potential gradients, and its significance for polar IAA transport.. New Phytol.

[30] Sahlin P, Soderberg B, Jonsson H (2009). Regulated transport as a mechanism for pattern generation: capabilities for phyllotaxis and beyond.. J Theor Biol.

[31] Reinhardt D, Pesce E. R, Stieger P, Mandel T, Baltensperger K (2003). Regulation of phyllotaxis by polar auxin transport.. Nature.

[32] Mitchison G. J (1977). Phyllotaxis and Fibonacci series.. Science.

[33] Douady S, Couder Y (1992). Phyllotaxis as a physical self-organized growth process.. Phys Rev Lett.

[34] Ditengou F. A, Teale W. D, Kochersperger P, Flittner K. A, Kneuper I (2008). Mechanical induction of lateral root initiation in Arabidopsis thaliana.. Proc Natl Acad Sci U S A.

[35] Williamson R. E (1990). Alignment of cortical microtubules by anisotropic wall stresses.. Aust J Plant Physiol.

[36] Peterson R. E (1974). Stress concentration factors: charts and relations useful in making strength calculations for machine parts and structural elements.

[37] Newell A. C, Shipman P. D, Sun Z (2008). Phyllotaxis: cooperation and competition between mechanical and biochemical processes.. J Theor Biol.

[38] de Reuille P. B, Bohn-Courseau I, Godin C, Traas J (2005). A protocol to analyse cellular dynamics during plant development.. Plant J.

[39] Shaner N. C, Lin M. Z, McKeown M. R, Steinbach P. A, Hazelwood K. L (2008). Improving the photostability of bright monomeric orange and red fluorescent proteins.. Nat Methods.

[40] Craft J, Samalova M, Baroux C, Townley H, Martinez A (2005). New pOp/LhG4 vectors for stringent glucocorticoid-dependent transgene expression in Arabidopsis.. Plant J.

