# Supplementary material for: Alignment between PIN1 Polarity and Microtubule Orientation in the Shoot Apical Meristem Reveals a Tight Coupling between Morphogenesis and Auxin Transport
Source: PLoS Biol. 2010 Oct 19;8(10):e1000516. doi: 10.1371/journal.pbio.1000516 (PMC2957402; doi:10.1371/journal.pbio.1000516)
Supplement: Text S1 — Biomechanical model details and stability analysis. (0.09 MB PDF) [file pbio.1000516.s001.pdf]

# 1 Model

Computer simulations were performed in a model representing an epidermal (L1) shoot tissue layer which combined mechanical and biochemical interactions on the level of single cell and cell wall compartments. We designed a three-dimensional finite element model which accurately reflected elastic properties and geometry of cells, and which was highly based on the model presented in Hamant et al. (2008). The model was limited to not including plastic growth and dividing cells. We assumed that the L1 layer is under tension coming from turgor pressure and interaction with inner tissue. This implies that anticlinal walls of cells are stretched in the plane of the layer and experience tensile stresses. The magnitude of those stresses depends on the exact geometry of the cells and the elastic properties of their walls. The walls of the cells despite being rigidly connected to walls of the neighbors remain associated with each cell. This means that a wall between two cells is represented as two compartments, for which elastic properties are independent. The stress perceived in each part of the wall depends on elasticity of both compartments of a wall segment and thus changes of elastic modulus in the wall belonging to one cell affects stress in the wall of the other, neighboring cell as well. We assume further that elastic properties of the cell walls are affected by auxin concentration in this cell. Therefore the proposed mechanism conveys a way of communicating the relative difference in auxin level between neighboring cells to their walls and membranes, such that higher stresses localize to the wall with lower auxin concentration. If we assume now that high stress in the wall positively simulates relocation of the PIN1 to this membrane we obtain a positive feedback loop between auxin concentration in the cell and transport of auxin to this cell, which can lead to formation of a pattern of auxin peaks in the simulations.

The set of equations describing the change of auxin level $a_i$ in the cell $i$ including production, degradation, passive transport and PIN1 dependent active efflux of auxin can be written as:

$$\frac{da_i}{dt} = c_a - d_a a_i + \sum_{k \in \mathcal{N}_i} D(a_k - a_i) + \sum_{k \in \mathcal{N}_i} (\mathcal{P}_{ki} h(a_k) - \mathcal{P}_{ik} h(a_i)), \qquad (1)$$

where $c_a$ is constant production, $d_a$ degradation, $D$ passive transport constant. We use the cell-based formulation from Sahlin et al. (2009), based on the chemiosmotic transport theory, and where the constants are in relation

to the permeability of the active transport term (e.g. $D \propto p_{aH}/p_{PIN}$, where $p_{aH}$ is the passive permeability of the protonated form of auxin and $p_{PIN}$ is the permeability of the PIN1 dependent efflux [Sahlin et al. 2009]). The influx mediator AUX1 is assumed to be symmetrically localized, and apoplastic diffusion is not taken into account. The active transport is saturated with auxin level described by a Michaelis-Menten function $h(a_i)$;

$$h(a_i) = \frac{a_i}{K_a + a_i}. \tag{2}$$

PIN1 is assumed to be in quasi-equilibrium and the functions $\mathcal{P}_{ij}$ describe the concentration of the PIN1 protein resulting from stress dependent cycling between cytosol and membrane in the wall separating cells $i$ and $j$;

$$\mathcal{P}_{ij} = \frac{Pf(s_{ij})}{1 + \sum_{k \in \mathcal{N}_i} f(s_{ik})}. \tag{3}$$

The function $f(s_{ij}) = f_{exo}(s_{ij})/f_{endo}(s_{ij})$ describing the response of auxin to stress can be seen as a combination of an increased exocytosis $(f_{exo}(s_{ij}))$ and/or a decreased endocytosis $(f_{endo}(s_{ij}))$ and we chose to use

$$f(s_{ij}) = k_2(s_{ij})^n, \tag{4}$$

where $k_2$ and $n$ are constants and $s_{ij}$ is the stress measure in the wall of cell $i$ neighboring cell $j$ and $P$ measures the total amount of the PIN1 in the cell. The auxin dependent elastic modulus of the walls of cell $i$ is given by

$$E(a_i) = E_{min} + \frac{(E_{max} - E_{min})k_3^m}{a_i^m + k_3^m}, \tag{5}$$

where $E_{min}$ and $E_{max}$ are expected minimal and maximal values of the wall elasticity respectively and $k_3$ and $m$ are constant parameters [Hamant et al. 2008]. In the simulations we used the set of parameters estimated from experimental measurements (for auxin transport rates), or previous models where applicable (Tab. 1) [Jönsson et al. 2006, Hamant et al. 2008, Sahlin et al. 2009].

# 2 Stability analysis

To perform stability analysis of the homogenous fixed state we consider small deviations of auxin concentrations $\varepsilon_i$ from the homogenous solution $a$

$$a_i = a + \varepsilon_i, \tag{6}$$

| $c_a$ | $d_a$ | $D$ | $K_a$ | $P$ | $k_2$ | $n$ | $k_3$ | $m$ | $E_{min}$ | $E_{max}$ |
|---|---|---|---|---|---|---|---|---|---|---|
| 0.0012 | 0.0012 | 0.00035 | 1.0 | 1.0 | 3.0 | 3 | 1.0 | 2 | $80MPa$ | $400MPa$ |

Table 1: Typical numerical values of the parameters used in the model of stress-dependent PIN1 cycling between cell and wall compartments and auxin transport between cells. See the text for description of the symbols.

and linearize the model equations with respect to it. We also assume a regular lattice of cells, so all the walls have the same unperturbed length $L_0$ and cross section $A_0$, and isotropic force on each wall $F$. In such case the strain of the composite wall becomes

$$e_{ij} = \frac{F/A_0}{E(a_i) + E(a_j)}, \tag{7}$$

and the stress perceived on the one side of the wall is

$$s_{ij} = \frac{F/A_0}{1 + \frac{E(a_j)}{E(a_i)}}. \tag{8}$$

Thus we can express the relation (3) as a function of auxin concentration in cells $i$, $j$ and $k \in \mathcal{N}_i$ (neighbors to cell $i$)

$$\mathcal{P}_{ij} = g(a_i, a_j, \{a_k\}_{k\in\mathcal{N}_i}) \equiv \frac{\tilde{f}(a_i, a_j)}{1 + \sum_{k\in\mathcal{N}_i} \tilde{f}(a_i, a_k)}, \tag{9}$$

where

$$\tilde{f}(a_i, a_j) = f\left(s_{ij}(a_i, a_j)\right) = k_2 F/A_0 \frac{E^n(a_i)}{(E(a_i) + E(a_j))^n}, \tag{10}$$

and since $E(a_j)$ is a decreasing function of auxin concentration in the neighboring cell ($a_j$), $f(s_{ij}(a_i, a_j))$ is an increasing function of the auxin concentration in the neighboring cell, and hence we can directly relate stability analysis of this model with the previously proposed concentration-based model [Jönsson et al. 2006, Sahlin et al. 2009], and the continued analysis follow the cell-based analysis in Sahlin et al. (2009).

Linearizing Eq.(1) we obtain

$$\frac{d\varepsilon_i}{dt} = \sum_{k\in\mathcal{N}_i} \left[ \mathcal{C}_1(\varepsilon_k - \varepsilon_i) - \mathcal{C}_2(\sum_{l\in\mathcal{N}_k} \varepsilon_l - \sum_{l\in\mathcal{N}_i} \varepsilon_l) \right] - d_a\varepsilon_i, \tag{11}$$

with constants with respect to $\varepsilon$, $\mathcal{C}_1$ and $\mathcal{C}_2$ defined as

$$\mathcal{C}_1 = D + \left.\frac{\partial h(a_i)}{\partial a_i}\right|_a g(a, a, \{a\}) - h(a) \left.\frac{\partial g(a_i, a_j, \{a_k\})}{\partial a_j}\right|_a + \tag{12}$$

$$h(a) \left.\frac{\partial g(a_i, a_j, \{a_k\})}{\partial a_i}\right|_a \tag{13}$$

$$\mathcal{C}_2 = -h(a) \left.\frac{\partial g(a_i, a_j, \{a_k\})}{\partial a_k}\right|_a . \tag{14}$$

By applying a Fourier transform with

$$\varepsilon_{\mathbf{k}} = \sum_i \varepsilon_i e^{-i\mathbf{k}\cdot\mathbf{x_i}}, \tag{15}$$

we diagonalize the linearized model equation and by defining the form factor $S(\mathbf{k})$ as

$$S(\mathbf{k}) = \sum_j e^{i\mathbf{k}\cdot\mathbf{e_j}}, \tag{16}$$

where $\mathbf{e_j}$ is a set of vectors from a cell center to the centers of its neighbors, we can write it in the form

$$\frac{d\varepsilon_{\mathbf{k}}}{dt} = \left[\mathcal{N}\mathcal{C}_1(S(\mathbf{k}) - 1) - \mathcal{N}^2\mathcal{C}_2 S(\mathbf{k})(S(\mathbf{k}) - 1) - d_a\right]\varepsilon_{\mathbf{k}} = \lambda_{\mathbf{k}}\varepsilon_{\mathbf{k}}, \tag{17}$$

where $\mathcal{N}$ is the number of cell neighbors. If the real part of any of the eigenvalues $\lambda_{\mathbf{k}}$ is positive, a small perturbation from the homogeneous state makes the system unstable, which is a requirement for spontaneous pattern creation. We see that the requirement for positive eigen vectors is equivalent in this case to

$$\frac{(\mathcal{C}_1 - \mathcal{N}\mathcal{C}_2)^2}{4\mathcal{C}_2} > d_a. \tag{18}$$

This means that high value of $d_a$, which is an auxin degradation constant, will destroy pattern forming capabilities of the model. The constant $\mathcal{C}_1$ includes $D$, the relative strength of the passive and PIN1 mediated auxin transport rates, and both $\mathcal{C}_1$ and $\mathcal{C}_2$ depend on the values and slopes of the stress feedback and auxin transport functions at the homogenous fixed point, as described in more detail in Sahlin et al. (2009).

The wave vectors corresponding to the largest eigenvalue belong to the set

$$\Omega = \left\{\mathbf{k} : S(\mathbf{k}) = \frac{1}{2}\left(\frac{\mathcal{C}_1}{\mathcal{N}\mathcal{C}_2} + 1\right)\right\}. \tag{19}$$

# References

[Jönsson et al. 2006] Jönsson H, Heisler M, Shapiro BE, Meyerowitz EM, Mjolsness E. 2006. An auxin-driven polarized transport model for phyllotaxis. *Proc Natl Acad Sci* **103**: 1633-1638.

[Hamant et al. 2008] O. Hamant, M. Heisler, H. Jönsson, P. Krupinski, M. Uyttewaal, P. Bokov, F. Corson, P. Sahlin, A. Boudaoud, E. M. Meyerowitz, Y. Couder, and J. Traas 2008. Developmental patterning by mechanical signals in Arabidopsis. *Science* **322**, 1650-1655.

[Sahlin et al. 2009] P. Sahlin, B. Söderberg, and H. Jönsson 2009. Regulated transport as a mechanism for pattern generation: Capabilities for phyllotaxis and beyond. *Journal of Theoretical Biology* **258**, 60-70.
